# Assessment of the capacity of a pyrophosphate-based mouth rinse to inhibit the formation of supragingival dental calculus. a randomized double-blind placebo-controlled clinical trial

**DOI:** 10.4317/medoral.23036

**Published:** 2019-08-19

**Authors:** Carla Fons-Badal, Rubén Agustín-Panadero, Mª Fernanda Solá-Ruíz, Francisco Alpiste-Illueca, Antonio Font-Fons

**Affiliations:** 1 DDS, PhD. Associate Professor, Department of Dental Medicine, Faculty of Medicine and Dentistry, University of Valencia, Valencia, Spain; 2 DDS, PhD, MD. Adjunct Professor, Department of Dental Medicine, Faculty of Medicine and Dentistry, University of Valencia, Valencia, Spain; 3 DDS, PhD, MD. Professor, Department of Dental Medicine, Faculty of Medicine and Dentistry, University of Valencia, Valencia, Spain

## Abstract

**Background:**

This study aimed to analyze the efficacy of an anti-calculus mouth rinse and its possible adverse effects on the mucosa and teeth.

**Material and Methods:**

This randomized double-blind placebo-controlled clinical trial included 40 patients with treated and managed periodontal disease, all with a history of rapid calculus formation. Patients used a pyrophosphate-based test mouth rinse (B) or a placebo (A). A range of parameters were measured for: saliva (saliva flow, pH and chemical composition); calculus (Volpe-Manhold [V-M] index, weight, and volume); adverse effects on mucosa and teeth; and the patients’ subjective perceptive of mouth rinse efficacy.

**Results:**

The test mouth rinse B produced reductions in urea, uric acid, and phosphorous, calcium, saliva flow, and increases in pH. V-M index and calculus weight decreased after using the test mouth rinse. Calculus volume decreased with both mouth rinses. No changes to the mucosa or teeth were observed. Patients perceived that the test mouth rinse was more effective.

**Conclusions:**

The test/B and placebo mouth rinses both modified certain parameters in saliva composition, particularly reductions in urea, uric acid, and phosphorous. Calcium tended to increase after using the test-B mouth rinse. The results did not demonstrate the anticalculus efficacy of the pyrophosphate-based mouth rinse or positive effects on saliva flow or composition. This field requires further research, as no product has been developed that prevents calculus formation completely.

** Key words:**Dental calculus, anticalculus mouth rinse, Volpe Manhold index.

## Introduction

Dental calculus is the consequence of the mineralization of bacterial plaque, which compromises oral health as it increases the accumulation of plaque and bacterial toxins and impedes their removal due to the surface roughness it produces. For this reason, calculus prevents the patient from maintaining an effective oral hygiene regime, which then facilitates the formation of further plaque (1).

The main periodontal diseases – gingivitis and periodontitis – have been related to the accumulation of dental plaque among other factors (2). Calculus accumulation varies across the general population. One group of individuals are known as ‘rapid calculus formers’ who, in spite of maintaining good plaque control, need frequent visits to the dentist to manage the rapid formation of supragingival calculus (1).

To deal with this problem, a large range of products have been marketed in recent years (mouth rinses, toothpastes, chewing gums, etc.). Numerous studies have shown that pyrophosphates, commonly used in these products, reduce the percentage of calculus formed (3-8). Nevertheless, in rapid calculus formers these products are not enough to deal with the problem. These patients continue to require frequent visits to the dentist to maintain adequate oral health. In this context, the results of trials of anti-calculus products are limited, suggesting the need for further research in this field (9).

The main aim of this study was to evaluate the magnitude of calculus formation after using a pyrophosphate-based anticalculus mouth rinse in order to assess its efficacy and possible adverse effects on the mucosa and teeth.

Specific objectives were to: 1. Measure the volume, pH, and chemical composition of saliva to determine whether use of the mouth rinse modified saliva and whether there was any relation between saliva modification and the quantity of calculus formed; 2. To assess the effects of the pyrophosphate-based mouth rinse on the bacterial plaque microorganisms associated with calculus formation (Eubacterium Saburreum, Corynebacterium matruchotii, Veillonella parvula, streptococcus salivarius, Streptococcus sanguis and Streptococcus mutans).

## Material and Methods

The present study, conducted at the Stomatology Department, Faculty of Medicine and Dentistry, at the University of Valencia (Spain), set out to study the efficacy of a pyrophosphate-based mouth rinse marketed by Dentaid (Barcelona, Spain) for inhibiting calculus formation among a group of rapid calculus formers.

This randomized, double-blind, placebo-controlled, clinical trial was approved by the University of Valencia Ethics Committee and fulfilled Declaration of Helsinki and European Council guidelines for research involving human subjects, as well Spanish legislation applying to biomedical research, data protection, and bioethics. All patients selected to participate presented (treated and managed) periodontal disease and a history of rapid calculus formation. Each patient was provided with full information about the study procedures and objectives and all signed an informed consent form.

Subjects used both the test mouth rinse (B) which contained pyrophosphate-based formula (0.85% Tetrasodium pyrophosphate decahydrate; 0.5% Sodium Hexametaphosphate; 0.5% Sodium Tripolyphosphate 0.50%; 0.05% Sodium Fluoride [226 ppm of F ions]) and a placebo mouth rinse (A) with the same composition as the test mouth rinse B but without the main active ingredients. Both had the same organoleptic properties so that neither subjects nor clinicians could distinguish between them. All patients used both mouth rinses throughout the study period, using the test product for a time and the placebo at another; this was designed to maximize sample size, given the difficulties of locating numbers of rapid calculus formers who also present good oral hygiene maintenance. The order in which the mouth rinses were used was randomized.

Initial supragingival scaling was performed, and then each mouth rinse was used for three months. Scaling and polishing were repeated between mouth rinses (test B / placebo A), when patients spent 24 hours without using any mouth rinse.

To ensure that patients had followed the mouth rinse regime correctly, a measured quantity of mouth rinse was delivered to each patient, providing a specific amount to cover each day of mouth rinse use; patients were asked to return any leftovers at the end of the 3-month period.

Throughout the study, all patients used the same toothbrush (Vitis® suave, Dentaid®, Cerdanyola, Barcelona, Spain) and the same toothpaste (Vitis® encías, Dentaid®).

Inclusion criteria were as follows: patients who had completed treatment for active periodontal disease; patients in the maintenance phase for at least 2 months before the start of the study; patients with a demonstrable history of rapid calculus formation; patients presenting stable clinical insertion levels and probing depth of 3 mm or less in the fifth sextant (at least); patients willing to adhere strictly to the study protocol. Exclusion criteria were: patients with missing teeth in the fifth sextant; the presence of systemic disorders that could modify saliva flow or have some repercussion for periodontal tissue; patients with poor plaque control (Silness and Löe plaque index >1 in test teeth); patients taking medication (antibiotics, anti-inflammatories, anti-depressives) or using mouth rinses (antiseptic or anticalculus mouth rinses) for 3 months before the start of the study; patients presenting localized factors that might influence plaque retention such as fillings, orthodontic apparatus, temporary or definitive splints/guards, fixed or removable prostheses.

A series of parameters were registered for saliva and calculus measurement to analyze the efficacy of the mouth rinses from different perspectives. Salivary parameters were: saliva flow in repose, measured using the drainage technique; pH, measured using a digital pH-meter (PCE Group, Albacete, Spain); and chemical analysis performed at a hospital center (Hospital La Fe, Valencia, Spain) to determine concentrations of urea, uric acid, calcium, phosphate, sodium, potassium and chlorine (two self-analyzers were used: 1. Modular Analytics SWA, Hitachi 917 [Roche®, Basle, Switzerland]; 2. BNII® DadeBehring [Siemens®, Berlin, Germany]).

Supragingival bacterial plaque analysis: a sample of bacterial plaque was harvested from the fifth sextant. Plaque was collected during all patient examinations, from the lingual and vestibular faces of the test teeth using a sterile curette and placing the sample in conservation solution (reduced transport fluid [RTF]) in an Eppendorf tube for later analysis. All samples were sent to a laboratory (Laboral, Barcelona, Spain) for analysis to determine the weight and volume of calculus and the presence of bacteria: Eubacterium saburreum, Corynebacterium matruchotii, Veillonella parvula, Streptococcus salivarius, Streptococcus sanguis and Streptococcus mutans.

The Volpe-Manhold index was used to measure the magnitude of calculus formation; it was weighed with analysis scales (PCE Group®, Albacete, Spain) and its volume measured with a pycnometer (Afora®, Barcelona, Spain). For measurement, the calculus was detached from the dental surface with an ultrasonic air scaler (SONICflex®, KaVo Dental, Fruehauf Drive, Charlotte, USA) and collected with a bone aspirator (Onmia,® Proclinic, Barcelona, Spain).

Lastly, possible changes to the oral mucosa and teeth were assessed visually.

Patients’ subjective perceptions of the efficacy of the mouth rinses were registered by means of a questionnaire.

Descriptive statistics of the parameters and differences between mouth rinses (placebo A and test B) were calculated: mean, standard deviation, minimum, maximum, median, and absolute and relative frequencies. Normal distribution of continuous measurements was checked with the Kolmogorov-Smirnov test. Inferential analysis was performed to determine the existence of significant differences in response parameters due to the mouth rinse used (placebo A/test B). A general linear model of repeated measures was estimated for response variables showing normal distribution. The efficacy of the test mouth rinse was assessed by means of the intra-subject interaction evaluated by Pillai’s trace value associated F-statistic. The Bonferroni test was used for post-hoc multiple comparisons to evaluate the changes to a parameter after using each of the mouth rinses. For other non-continuous indicators, or those that did not show normal distribution, the influence of the mouth rinses was evaluated by means of McNemar’s test (for changes in proportion) or the Wilcoxon test for paired samples (for changes in distribution). Statistical significance was set at 5% (α=0.05).

## Results

This randomized double-blind placebo-controlled clinical trial assessed the efficacy of a pyrophosphate-based mouth rinse for inhibiting dental calculus in a sample of ‘rapid calculus forming’ patients. Three patients were lost during the study period as they did not fulfill the study protocol, leaving a total sample of 37 subjects (n=37). All patients used both mouth rinses (test B and placebo A), so a single group constituted both control group (placebo A) and test group (test mouth rinse B).

In the statistical tests applied to the data to detect differences induced by the mouth rinses, for a paired t test with 5% significance level, considering an effect size of 0.5, the power reached was 0.84.

-Saliva Parameters 

The two mouth rinses presented very similar patterns in their effects on saliva composition. Sodium, chlorine and potassium levels were unaffected by both mouth rinses and remained stable throughout the study. Urea, uric acid and phosphorous decreased similarly with the use of both mouth rinses. Calcium, saliva flow, and pH tended to increase more with mouth rinse test-B. For some parameters such as calcium and saliva flow, test-B intensified differences in final values compared with placebo-A. For sodium, this tendency was reversed ( Table 1).

**Table 1:**
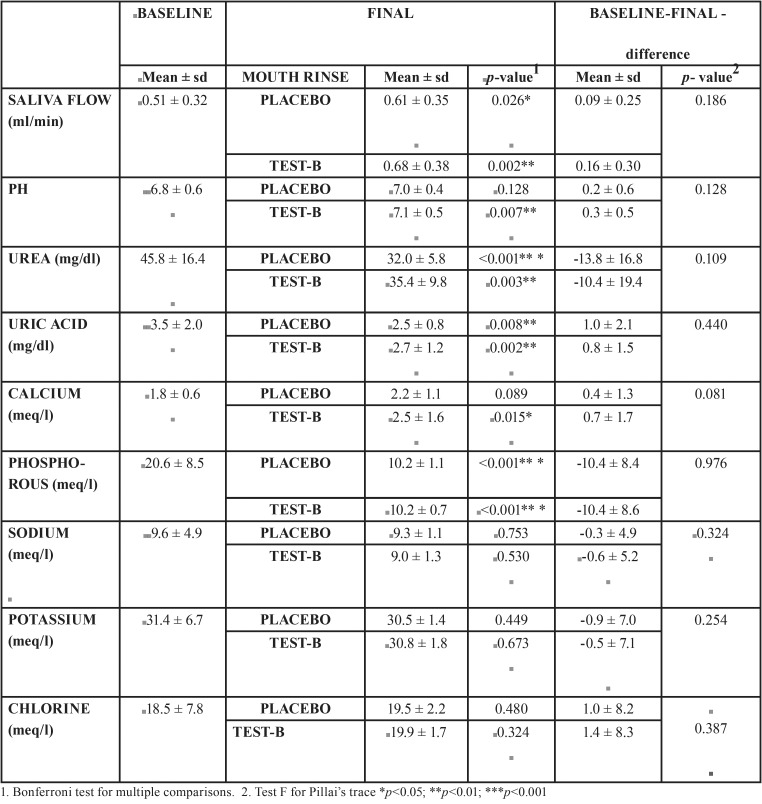
Saliva Parameters: Results obtained from GLM of repeated measures with intra-subject factors: time and mouth rinse; and inter-subject factor: the order in which mouth rinses were used.

-Calculus dimension parameters 

Baseline mean Volpe-Manhold index was 19.70 ± 9.62, decreasing to 13.7 ± 7.1 units after placebo-A use and 11.0 ± 6.5 units after use of the test-B mouth rinse. The reduction obtained with test-B mouth rinse was statically significant (*p*<0.001) but so was the reduction achieved with the placebo-A (*p*<0.001). So no definitive evidence was found for a reduction in calculus due to an exclusive effect of the test-B mouth rinse. Nevertheless, the magnitude of the reduction was not equal with both mouth rinses, whereby the test-B mouth rinse produced a significantly larger reduction than the placebo-A (*p*=0.012) ( Table 2).

**Table 2:**
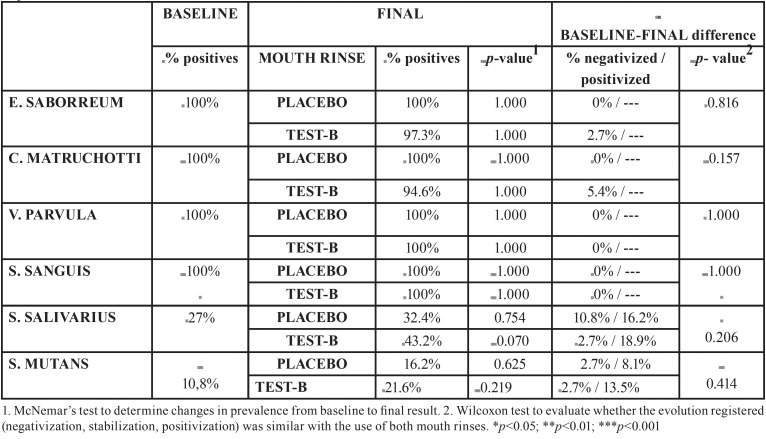
Calculus parameters: Results obtained from GLM of repeated measures with intra-subject factors: time and mouth rinse; and inter-subject factor: the order in which mouth rinses were used.

Calculus weight was analogous to volume in the reductions obtained by the two mouth rinses. Mean weight at baseline was 21.9 + 13.2 mg decreasing to 15.1 ± 10.7 mg with use of the placebo-A, and to 10.8 ± 9.6 mg after using the test-B mouth rinse. Both reductions were statistically significant (placebo-A: *p*<0.001; test-B: *p*<0.001;), although the effect of test-B was of greater relevance (*p*=0.004).

Mean volume at baseline was 21.9 ± 12.3 mm3, decreasing to 14.2 ± 9.7 mm3 after using placebo-A and 14.3 ± 14.9 mm3 after using the test-B mouth rinse. The mean decreases with both mouth rinses were statistically significant (placebo-A: *p*<0.001; test-B: *p*=0.009;). Nevertheless, both reductions were significantly equal (*p*=0.989).

-Supragingival bacterial plaque analysis 

Both qualitative and quantitative polymerase chain reaction (PCR) analyses were performed to determine the presence or absence of bacteria and the bacterial load.

-PCR analysis

No significant change in prevalence (positivity) was produced in any of the bacteria after using the either the placebo-A or test-B mouth rinses. The only tendency of note was the increase registered for S. Salivarius after using the test-B mouth rinse (*p*=0.070). At baseline, 27% of patients showed positive, while after test-B use the percentage rose to 43.2% ( Table 3).

**Table 3:**
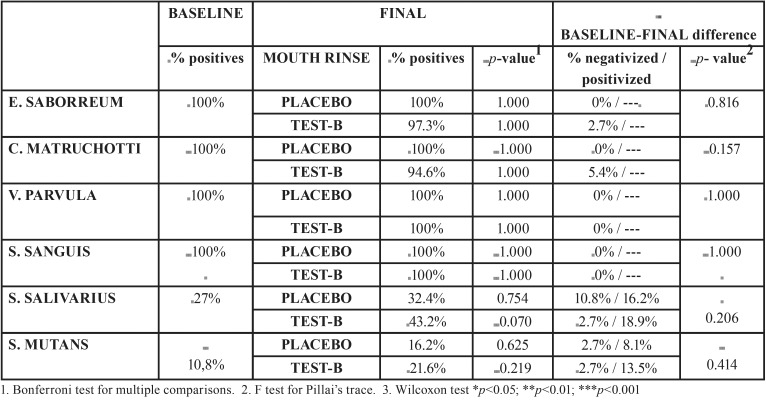
Bacteriological parameters: prevalence.

-Bacterial load (qPCR) 

This analysis was performed only in those patients who presented a positive PCR and qPCR over the detection limit. In order to make data simpler to analyze, CFU (colony forming units) load was transformed into a decimal logarithm scale, making data more manageable. In addition, providing the sample size is adequate, logarithmic transformation favors normalization of the original variables, stabilization of their variance, and parametric testing ( Table 4).

**Table 4:**
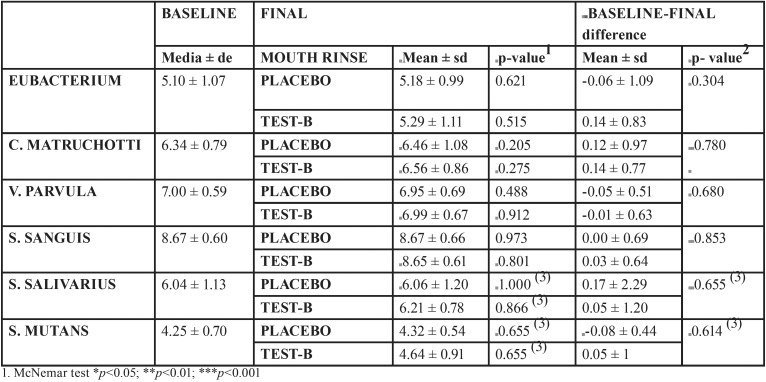
Bacterial parameters: transformed decimal logarithm scale of CFU load. Results obtained from GLM of repeated measures with intra-subject factors: time and mouth rinse; and inter-subject factor: the order in which mouth rinses were used for the bacteria: Eubacterium, Matruchotti, V. parvula and S. sanguis. Results of Wilcoxon test for S. salivarius and S.mutans. Baseline: patients above detection level qPCR.

In terms of both prevalence and absolute bacterial load, neither mouth rinse appeared to induce relevant variations in the bacterial species analyzed.

-Product tolerance parameters 

Under clinical examination, no anomalies were observed in photographs taken at different stages of the study. Three patients reported canker sores after using the test-B mouth rinse and one after using the placebo, but also mentioned that they occasionally suffered these sores when not using mouth rinses. In the same way, some patients reported staining but these individuals also underwent staining from time to time regardless of mouth rinse use; staining was not seen to increase or decrease as a result of the study mouth rinses. The mouth rinses did not appear to have any effects on hard tissues or the oral mucosa.

-Subjective perception of mouth rinse efficacy 

The questionnaire to assess the efficacy of the mouth rinse for preventing calculus formation found that 51.3% of patients reported forming less calculus than normal with the test-B mouth rinse, while 8.1% reported less calculus with the placebo-A, and 37.8% found both mouth rinses effective for preventing calculus formation; 2.7% said that neither mouth rinse was effective ( Table 5).

**Table 5:**
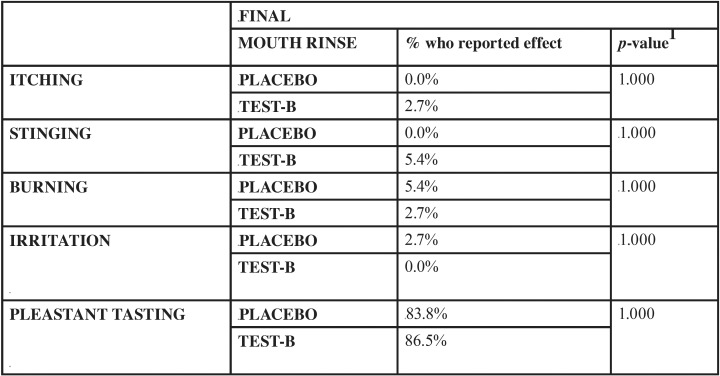
Subjective perception parameters: frequency of reactions.

## Discussion

The present study investigated a sample of 37 patients (three were lost through failure to fulfill the study protocol). This number was sufficient to perform statistical analysis and detect significant differences between the mouth rinses assayed, reaching a statistical power of 0.84. Most similar studies have had larger sample sizes, although in some, the level of oral hygiene maintenance among the rapid calculus formers was not checked (10), and in others the sample consisted of all kinds of patients rather than rapid calculus formers exclusively (3,7). It was not easy to recruit numbers of patients who were rapid calculus formers and also maintained good plaque control, but these criteria constituted an important characteristic of the present study. Oral hygiene is usually linked to the amount of calculus formed, so the combination of good plaque control and major calculus formation is unusual.

To measure calculus quantity, the methods used in previous research were identified and assessed through an extensive literature search. There are various indices used for measuring calculus: the Volpe-Manhold index, the marginal line calculus index, the calculus surface index, etc. Among these methods, the Volpe-Manhold is the most frequently employed in trials of anticalculus products (3,5,7,11-13). In addition, the present study measured both the weight and volume of calculus, a novel feature not found in previous studies with similar characteristics.

The method used for collecting calculus was similar to that used in a study by Grases *et al.* (14), although in the earlier work calculus underwent chemical analysis and was not quantified as in the present study.

Calculus measurement was performed on six lower anterior teeth as this is the area where the most supragingival calculus is deposited (15,16) and previous trials of anticalculus products have focused on this area (3,5,15,16).

As for the duration of mouth rinse use, in most other studies the time passed between the start of the study period and data collection – varying between 3 weeks and 2 months – has been shorter than we consider necessary (3,5,7,8,14,17). But some tested the mouth rinses for a period of 3 months as in the present study (7,18,19). The reason for the 3-month period derives from the fact that this is the maximum time lapse between the periodontal support sessions that rapid calculus formers require. In this way, it was possible to determine whether the mouth rinse was effective, and would allow patients longer intervals between dental check-ups.

In the present study, saliva flow per minute was found to increase from 0.51 ± 0.32 ml/min at baseline to 0.61 ± 0.35 ml/min after placebo-A use, and to 0.68 ± 0.38 ml/min after test-B mouth rinse use. In comparison with previous studies, although some increase was observed in the present study, this was nowhere near the values obtained for stimulated saliva flow (20).

The increase in pH after using the test-B mouth rinse was statistically significant compared with baseline level (*p*=0,007), an effect that was not produced by the placebo-A mouth rinse (*p*=0.128). This increase in pH was probably due to the increased saliva flow also produced. Dawes reported that the pH of saliva stimulated by chewing gum was significantly higher than non-stimulated saliva (16). In the present study no relation was found between saliva pH and increased calculus deposits. On the contrary, according to the literature, calculus deposits are more related to the pH of bacterial plaque than salivary pH (21,22).

The chemical values obtained fell within the range considered normal. It is of note that calcium, although within the range of normality, increased in concentration after the use of both mouth rinses, especially the test-B mouth rinse. Mandel (2) affirmed that calcium in submaxillary saliva is significantly more abundant in rapid calculus formers than patients who are not, while Dawes noted that as the saliva flow rate increases, so do the mean concentrations of sodium and calcium (23).

Regarding the somewhat inconclusive results for salivary parameters, not all studies have established a direct relation between saliva components and calculus formation and other lines of investigation place more importance on the chemical components of bacterial plaque than those of saliva (24,25).

As for calculus quantification parameters, the mean baseline Volpe-Manhold index was 19.70 ± 9.62, decreasing to 11.0 ± 6.5 after using test B mouth rinse, and 13.7 ± 7.1 after the placebo-A. So a statistically significant reduction was found for both the test-B and the placebo-A mouth rinse, the decrease being greater with the test product (reduction of 44.1%). Mallatt *et al.* (3) obtained a reduction in Volpe-Manhold index of 26% for a test mouth rinse (test 4.74; control 6.40). These Volpe-Manhold scores were lower as the subjects were not rapid calculus formers and the study duration was shorter. Gaengler *et al.* (5) registered a decrease in calculus of 25.5% with the use of a product compared with a placebo, while Llena *et al.* (7) registered a smaller decrease than the present study between baseline and final values (baseline 12.39; placebo 10.95; and test 9.41).

Regarding calculus weight and volume, weight was seen to reduce significantly after using the test-B mouth rinse compared to baseline values and the placebo-A; nevertheless, volume behaved similarly with both mouth rinses, although values did improve in comparison with baseline measurements. The fact that weight and volume did not correlate may be explained by the lower density of calculus after using the test-B mouth rinse.

It can be seen that using the test mouth rinse produced a statistically significant decrease in calculus, but so did the placebo, although not of the same magnitude. This finding suggests that the reduction in calculus was not due to the mouth rinse exclusively. It could be a result of a boost in motivation by patients in response to participation in a study. This is known as the Hawthorne effect: a modification of behavior among subjects when they know they are being monitored. In the present case, patients had been attending three-monthly oral hygiene check-ups for years to stabilize periodontal disease and had demonstrated a history of good oral hygiene (plaque index scores of 1 or below). Nevertheless, the placebo also had a beneficial effect on calculus reduction, suggesting that perhaps plaque control maintained by the patients before the start of the study was not as good as we were led to believe, and so oral hygiene practices might have improved during the study period leading to reductions in calculus formation during placebo use.

No changes to oral mucosa or hard tissues were observed, a finding that concurs with the literature, which does not report any adverse effects derived from pyrophosphates (3,18,26-28).

## Conclusions

1. Both the test-B and the placebo-A mouth rinses modified certain parameters in the chemical composition of saliva, particularly reductions in urea, uric acid, and phosphorous. Calcium tended to increase with the test-B mouth rinse. Saliva flow and pH levels increased after use of the test-B mouth rinse.

2. Bacterial analysis observed a tendency for reductions in the presence of bacteria after using the mouth rinses, but neither the prevalence nor the bacterial load underwent statistically significant changes.

3. Oral mucosa and teeth were unaffected by the use of either mouth rinse.

4. The pyrophosphate-based mouth rinse was not shown to have anticalculus effects or any positive effects on saliva flow or saliva composition.

5. Further research is required in this field as to date no product has been developed that completely prevents the formation of calculus.
